# Research Note: Genome-wide association study identifies a 1.03-Mb resistance-associated haplotype on chromosome 12 associated with avian leukosis virus susceptibility in Wuhua yellow chickens

**DOI:** 10.1016/j.psj.2026.107246

**Published:** 2026-06-10

**Authors:** Xunhe Huang, Tingting Xie, Yongjie Xu, Zhuoxian Weng, Li Zhang, Bingwang Du

**Affiliations:** a Guangdong Provincial Key Laboratory of Conservation and Precision Utilization of Characteristic Agricultural Resources in Mountainous Areas, Guangdong Innovation Centre for Science and Technology of Wuhua Yellow Chicken, School of Life Sciences, Jiaying University, Meizhou, 514015, China; b College of Coastal Agricultural Sciences, Guangdong Ocean University, Zhanjiang, 524088, Guangdong, China

**Keywords:** Genome-wide association study, Avian leukosis virus, Wuhua yellow chicken, Genetic susceptibility, Novel candidate gene

## Abstract

Avian leukosis virus (ALV) causes immunosuppression and tumors, resulting in considerable economic losses in the poultry industry. However, the genetic basis of ALV susceptibility in indigenous breeds remains elusive. Herein, we conducted a genome-wide association study for ALV susceptibility in 259 Wuhua yellow chickens, an indigenous Chinese breed with known disease resistance. ALV infection status was determined by p27 antigen ELISA at 46 weeks of age, with 10 individuals (3.9%) classified as positive. We identified 58 significant SNPs, all located on chromosome 12 and concentrated within a 1.03-Mb region (12.19–13.22 Mb), using SAIGE to account for extreme case‑control imbalance. Gene annotation demonstrated 11 candidate genes, among which *FHIT* accounted for 44 of the 58 significant SNPs (76%). Additionally, *PDHB, PTPRG, FAM3D*, and *FAM107A* were identified as potential candidate genes associated with ALV susceptibility. Notably, conditional analysis confirmed that the lead SNP in *FHIT* (chr12:12,650,715) is the main driver of this cluster, given that its inclusion as a covariate eliminated all neighboring signals. Functional enrichment showed the citrate cycle as the most considerably enriched pathway, highlighted by *PDHB*, alongside pyruvate and carbon metabolism. The convergence of tumor suppression (*FHIT, FAM107A*), metabolic reprogramming (*PDHB*), and immune/signaling (*PTPRG, FAM3D*) genes within a single genomic region suggests a potential resistance-associated haplotype block. Our study provides promising candidate genetic markers for breeding ALV-resistant lines and offers novel insights into host–retrovirus interactions beyond traditional immune signaling.

## Introduction

Avian leukosis (**AL**) is a neoplastic and immunosuppressive disease caused by avian leukosis virus (**ALV**), which imposes substantial economic losses on the global poultry industry. Multiple subgroups (A–K) have been identified based on viral envelope properties and host range, with subgroup J (**ALV-J**) being especially detrimental owing to its broad tropism and high pathogenicity (Fandino et al., 2023; Tan et al., 2024). Although eradication programs have successfully decreased exogenous ALV infection in commercial lines, the virus remains widespread in Chinese indigenous breeds and free-range flocks, posing a continuous re‑emergence threat (Fandino et al., 2023). Advances in genome-wide association studies (**GWAS**) have facilitated substantial progress in identifying genes linked to ALV susceptibility, such as IFN-α/β in Qingyuan partridge chickens (Jie et al., 2025) and immune-regulatory genes on chromosome 6 in Chengkou Mountain chickens (Li et al., 2025). However, the genetic basis of chronic-phase tumor suppression and metabolic adaptation remains elusive.

The Wuhua yellow chicken, a traditional breed native to Guangdong Province, China, represents an unselected local genetic resource with high genetic diversity and natural disease resistance traits (Weng et al., 2020). Previous genomic studies have shown substantial selective sweeps in immune-related pathways in this breed; however, its response to viral infections at the whole-genome level has not been characterized (Weng et al., 2020). Standard GWAS can produce inflated type I error rates, given the extreme case–control imbalance often encountered in natural populations. The SAIGE method effectively addresses this limitation by implementing a logistic mixed model with saddlepoint approximation (Zhou et al., 2018).

Here in, we conducted the first GWAS in Wuhua yellow chickens to identify novel ALV susceptibility loci. We identified a 1.03-Mb haplotype block on chromosome 12—anchored by the tumor suppressor *FHIT* and supported by potential novel candidates *PDHB, PTPRG, FAM3D*, and *FAM107A*. This block can serve as a promising target for molecular breeding, by integrating conditional analysis and functional enrichment.

## Materials and methods

### Ethics statement

The Institutional Animal Care and Use Committee of Jiaying University approved all animal experiment procedures (Protocol No. JYYXLL2025-13).

### Sample collection and phenotyping

A total of 259 Wuhua yellow chickens from the same population that was previously described (Huang et al., 2025) were employed. Blood samples were collected when the chickens were 46 weeks of age and centrifuged under low‑temperature conditions. The serum was separated under aseptic conditions. The upper serum layer was subsequently transferred to DF‑1 cell suspension for co‑culture. Finally, ALV infection status was determined by detecting the p27 antigen using a commercial ELISA kit (Guangdong Biaoyun Biotechnology Co., Ltd., Yunfu, China) following the manufacturer’s protocol. Samples with a sample-to-positive (S/P) ratio of > 0.2 were defined as being positive.

### Genotyping and quality control

The raw whole-genome sequencing data for the 259 Wuhua yellow chickens were derived from our previously study (Huang et al., 2025); they are publicly accessible under NCBI BioProject PRJNA1288062. Briefly, genomic DNA was extracted from blood samples and subjected to high-throughput sequencing on the DNBSEQ-T7 platform (Annoroad, China) to generate 150-bp paired-end reads. The raw data were processed with fastp v0.23.4 and aligned to the chicken reference genome GRCg6a using BWA‑MEM v0.7.17. Variant calling followed GATK v4.2.6 best practices. SNPs were filtered with vcftools v0.1.16 (parameters: –min‑alleles 2, –max‑alleles 2, –maf 0.05, –geno 0.9, –hwe 1e-6). After quality control, genotype refinement was conducted using Beagle v5.4 with default parameters and an effective population size of 10,000, using the study samples as a reference panel. Linkage disequilibrium (**LD**) pruning was conducted with PLINK v1.90 (–indep‑pairwise 50 10 0.1) to obtain a set of independent SNPs.

### Genome‑wide association study

Principal component analysis was performed with PLINK using the pruned SNPs. We performed association analysis using SAIGE v1.3.1 (Zhou et al., 2018) to account for the extreme case–control imbalance. Using the pruned SNPs, a genomic relationship matrix (**GRM**) was calculated. Single‑variant association tests were performed with the GRM included as a random effect and the first three principal components as fixed effects. Significance thresholds were set at *P* < 0.05/*N*_indep_ (genome‑wide) and *P* < 1/*N*_indep_ (suggestive), where *N*_indep_ is the number of independent SNPs following LD pruning. Manhattan and Q‑Q plots were generated with the R package CMplot. SNP annotation was conducted with ANNOVAR v20240219. LD block analysis was conducted using LDBlockShow v1.40. Conditional analysis was performed using SAIGE by including the most significant SNP as a covariate in the model to assess whether other signals in the region remained independent.

### Odds ratio and post-hoc power analysis

Odds ratios (**OR**s) and 95% confidence intervals (**CIs**) were directly obtained from the SAIGE logistic mixed model as OR = exp(β) and 95% CI = exp(β ± 1.96 × SE), where β and SE are the effect size and standard error for the alternate allele, respectively. Post-hoc power was calculated with G*Power using α = 0.05, observed risk allele frequencies, and sample sizes.

### Functional enrichment analysis

For Gene Ontology (**GO**) and Kyoto Encyclopedia of Genes and Genomes (**KEGG**) enrichment analyses, candidate genes were submitted to KOBAS‑i (http://bioinfo.org/kobas). Terms with Benjamini–Hochberg corrected *P* < 0.05 were considered statistically significant.

## Results and discussion

### Genome-wide significant loci and FHIT’s dominant role

**Fig. 1:**
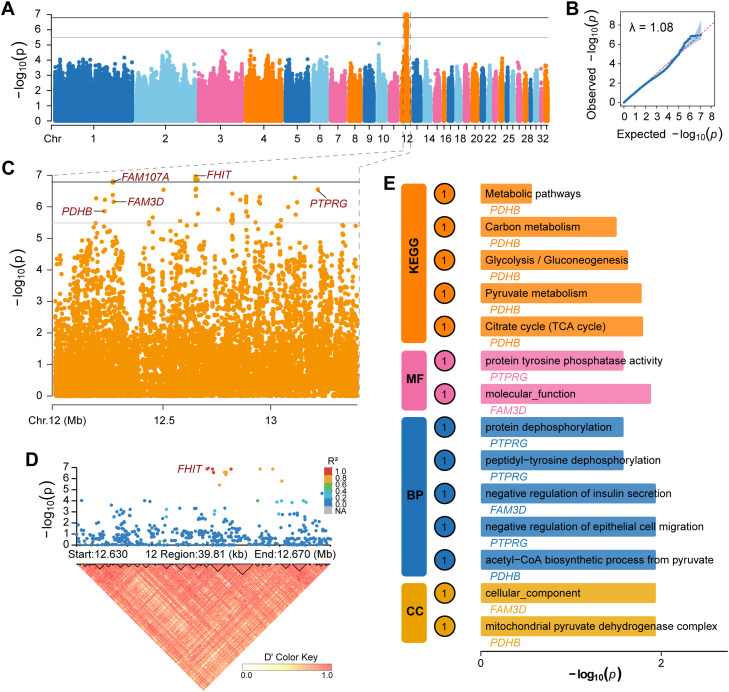
Genome-wide association study for ALV susceptibility in Wuhua yellow chickens. (A) Manhattan plot across all chromosomes. Each point represents an SNP. The black solid line indicates the genome-wide significance threshold (*P* < 1.62 × 10**^−^**^7^), and the grey solid line indicates the suggestive significance threshold (*P* < 3.24 × 10**^−^**^6^). (B) Quantile-quantile plot of the observed versus expected **−**log_10_(*P*) values. The genomic inflation factor (λ = 1.08) is indicated. (C) Regional plot of chromosome 12 (12.0–13.4 Mb) revealing the significant SNP distribution. Key candidate genes discussed in the text are labeled at their most significant SNP positions. (D) Linkage disequilibrium heatmap of the *FHIT* region centered on the lead SNP (chr12:12,650,715). (E) Enrichment bar plot for the Kyoto Encyclopedia of Genes and Genomes (KEGG) pathways and Gene Ontology (GO) terms. Colors indicate the three GO functional categories: biological process (BP), cellular component (CC), and molecular function (MF). The top relevant terms from each category are shown. Bar length represents –log_10_(*p*) value; the numbered circles indicate gene count.

Of the 259 chickens, 10 individuals (3.9%) were classified as being ALV-positive and 249 (96.1%) as negative. After quality control, 11,325,480 SNPs were retained for GWAS, with 308,857 independent SNPs identified following LD pruning. These independent SNPs were utilized to calculate the significance thresholds. The SAIGE analysis identified 58 significant SNPs (*P* < 3.24 × 10^−6^), all located on chromosome 12, of which 5 SNPs reached genome‑wide significance (*P* < 1.62 × 10^−7^) (Fig. 1A). The genomic inflation factor (λ = 1.08) indicated no population stratification (Fig. 1B). These SNPs clustered within a 1.03-Mb region (12,190,165–13,218,625 bp), revealing this interval as the main susceptibility locus.

Gene annotation showed that *FHIT* (fragile histidine triad diadenosine triphosphatase) is the predominant candidate gene, accounting for 44 of the 58 significant SNPs (76%) (Fig. 1C; Table 1). The lead SNP chr12:12,650,715 is an intronic variant in *FHIT* (*P* = 1.08 × 10^−7^; OR = 22,082), a tumor suppressor involved in DNA damage response, apoptosis, and p53 pathway crosstalk (Karras et al., 2014). *FHIT* disruptionis frequently noted in virus-associated cancers, including HPV- and HTLV-1-related malignancies. The extreme odds ratio reflects the marked allele frequency difference (cases 0.4 vs. controls 0.04) but should be interpreted with extreme caution owing to the small number of cases (*n* = 10) and the quasi-separation of alleles. This finding indicates that the exact magnitude is imprecisely estimated. Nevertheless, the saddlepoint approximation in SAIGE effectively controls for type I errors under such extreme case–control imbalances, ensuring that the identification of this novel locus is statistically robust and providing valuable targets for further investigation. The LD analysis revealed multiplex blocks within the region, with the lead SNP in an 8‑SNP block (0.60 kb) and larger blocks within *FHIT* (Fig. 1D), supporting a coordinated haplotype cluster. We performed conditional analysis to confirm whether this signal was the primary driver of the entire cluster. After adjusting for the lead *FHIT* SNP, all other significant associations in the region—including those for *PDHB, FAM107A, FAM3D*, and *PTPRG*—completely disappeared (*P_cond* of approximately 1.0). This fingding provides robust statistical evidence that a single QTL anchored by *FHIT* is the primary signal underlying the chromosome 12 association.

**Table 1:** Significant SNPs linked to ALV susceptibility on chromosome 12 in the Wuhua yellow chickens.

Chr	Position	P-value	Candidate Gene	OR (95% CI)	Power	Feature
12	12,650,715	1.08 × 10^−7^	*FHIT*	22,082 (551–884,041)	0.91	Intronic
12	13,112,193	1.19 × 10^−7^	ENSGALG00000056131	18,446 (486–699,763)	0.91	Intergenic
12	12,267,943	1.55 × 10^−7^	*FAM107A*	11,099 (342–360,140)	0.89	Intronic
12	13,218,625	2.81 × 10^−7^	*PTPRG*	6,087 (219–169,336)	0.89	Intronic
12	12,502,649	2.87 × 10^−7^	ENSGALG00000065207	1,394 (88–22,146)	0.92	Intronic
12	12,190,165	5.34 × 10^−7^	ENSGALG00000048013	999 (67–14,872)	0.91	Exonic
12	12,230,781	5.97 × 10^−7^	ENSGALG00000063924	18,991 (397–908,549)	0.84	Intronic
12	12,270,961	6.92 × 10^−7^	*FAM3D*	5,492 (183–164,589)	0.89	Downstream
12	13,081,102	1.01 × 10^−6^	ENSGALG00000054612	524 (43–6,456)	0.96	Intergenic
12	12,228,685	1.37 × 10^−6^	*PDHB*	265 (28–2,557)	0.94	Intronic
12	12,435,209	3.05 × 10^−6^	ENSGALG00000050643	479 (36–6,398)	0.93	ncRNA intronic

Note: Odds ratios (OR) should be interpreted with extreme caution owing to the small number of cases (*n* = 10) and quasi-separation of the alleles. Post-hoc power for the SNPs indicated ranged from 0.84 to 0.96. These high estimates reflect the large effect sizes noted under the limited sample size and should be interpreted accordingly.

### Novel candidate genes: metabolic, signaling, and immune-related candidates within a resistance-associated haplotype

Beyond *FHIT*, this study revealed multiple potential candidate genes that have not been previously associated with ALV susceptibility. Functional enrichment analysis of the 11 candidate genes highlighted metabolic pathways such as the citrate cycle, pyruvate metabolism, and carbon metabolism (Table 1, Fig. 1E). Four KEGG pathways were significantly enriched (corrected *P* < 0.05): citrate cycle, pyruvate metabolism, glycolysis/gluconeogenesis, and carbon metabolism—all highlighted by the identification of *PDHB* (pyruvate dehydrogenase E1 subunit beta) (chr12:12,228,685; *P* = 1.37 × 10^−6^; OR = 265), which encodes the E1β subunit of pyruvate dehydrogenase and regulates glycolytic flux into the TCA cycle (Gray et al., 2014). This finding is consistent with the fact that ALV-J infection is known to exploit host cellular metabolism to fuel rapid viral replication, a phenomenon similar to the Warburg effect in cancer cells (Xu et al., 2024). *PDHB* identification suggests that genetic variation in pyruvate-to-acetyl-CoA conversion may affect how efficiently the host can restrict viral metabolic hijacking.

Additionally, we identified *FAM107A, FAM3D*, and *PTPRG* as potential novel candidate genes within this cluster (Table 1). FAM107A inhibits cell invasion by modulating the actin cytoskeleton. Notably, FAM3D is a cytokine-like protein involved in mucosal immunity and inflammation. Given that ALV-J targets various tissues including the digestive and reproductive tracts, the inclusion of *FAM3D* as a potential candidate indicates that localized immune modulation may additionally play a role in host resistance. PTPRG is a known tumor suppressor that regulates cell signaling, and its expression is frequently downregulated in different malignancies. PTPG as a signaling regulator may interfere with the oncogenic signaling pathways initiated by ALV integration. The co-localization of these genes suggests that the Wuhua yellow chicken has a unique “resistance haplotype” encompassing tumor suppression (*FHIT* and *FAM107A*), metabolic efficiency (*PDHB*), signaling (*PTPRG*), and immune modulation (*FAM3D*).

### Comparison with other breeds and study limitations

The genetic architecture identified in this study varies from the interferon-focused loci found in Qingyuan (Jie et al., 2025) and Chengkou (Li et al., 2025) chickens. This may be owing to the breed’s unique selection history or the sampling timing (46 weeks), which captures markers of chronic oncogenesis rather than acute-phase immune response. The main limitation is the small number of ALV-positive individuals (*n* = 10), resulting in extremely large ORs ratios and imprecise effect sizes. While the results indicate an extremely strong association, the magnitude should be interpreted with caution. Despite this limitation, the strict statistical framework supports the validity of these potential associations. Additionally, although DF-1 co-culture confirmed ALV, low p27 S/P values (0.2–0.6) resulted in viral loads that were too low to enable subgrouping of ALV. This low viral load likely reflects chronic-phase (46 weeks) suppression driven by host immunity or viral escape. Furthermore, although conditional analysis pinpointed *FHIT*, the high LD in this 1.03-Mb region makes it challenging to definitively exclude the functional contributions of neighboring novel genes.

Future studies with larger samples and functional validation—such as CRISPR/Cas9-mediated knockout of *FHIT* or *PDHB*—are required to confirm causality. In future research, incorporating analysis during the peripuberty period of this breed (e.g., 18–19 weeks of age) to capture peak viral loads will be necessary to achieve successful viral isolation and multi-subgroup tracking. The results could help further determine whether these loci affect acute infection or chronic tumorigenesis. From a breeding perspective, the lead SNP in *FHIT* and the associated haplotype provide high-value targets for marker-assisted selection, providing a practical strategy to develop ALV-resistant Wuhua yellow chicken lines while preserving valuable indigenous genetic resources.

## CRediT authorship contribution statement

**Xunhe Huang:** Writing – review & editing, Writing – original draft, Visualization, Project administration, Methodology, Investigation, Funding acquisition, Formal analysis, Data curation, Conceptualization. **Tingting Xie:** Writing – review & editing, Resources, Methodology, Funding acquisition, Formal analysis, Data curation, Conceptualization. **Yongjie Xu:** Writing – review & editing, Resources, Data curation. **Zhuoxian Weng:** Writing – review & editing, Resources, Data curation. **Li Zhang:** Writing – review & editing, Conceptualization. **Bingwang Du:** Writing – review & editing, Conceptualization.

## Disclosures

The authors declare that they have no known competing financial interests or personal relationships that could have appeared to influence the work reported in this paper.
